# A cost minimisation and Bayesian inference model predicts startle reflex modulation across species

**DOI:** 10.1016/j.jtbi.2015.01.031

**Published:** 2015-04-07

**Authors:** Dominik R. Bach

**Affiliations:** a Department of Psychiatry, Psychotherapy, and Psychosomatics, University of Zurich, Switzerland; b Wellcome Trust Centre for Neuroimaging, University College, London, UK

**Keywords:** Motivational priming, Emotion, Fear

## Abstract

In many species, rapid defensive reflexes are paramount to escaping acute danger. These reflexes are modulated by the state of the environment. This is exemplified in fear-potentiated startle, a more vigorous startle response during conditioned anticipation of an unrelated threatening event. Extant explanations of this phenomenon build on descriptive models of underlying psychological states, or neural processes. Yet, they fail to predict invigorated startle during reward anticipation and instructed attention, and do not explain why startle reflex modulation evolved. Here, we fill this lacuna by developing a normative cost minimisation model based on Bayesian optimality principles. This model predicts the observed pattern of startle modification by rewards, punishments, instructed attention, and several other states. Moreover, the mathematical formalism furnishes predictions that can be tested experimentally. Comparing the model with existing data suggests a specific neural implementation of the underlying computations which yields close approximations to the optimal solution under most circumstances. This analysis puts startle modification into the framework of Bayesian decision theory and predictive coding, and illustrates the importance of an adaptive perspective to interpret defensive behaviour across species.

## Highlights

• We modelled costs of a blow to an organism׳s head, and of a protective startle response.
• Cost-minimising startle response increases as a blow becomes more likely.
• Fear-potentiated startle is predicted by Bayesian inference on blow probability.
• Startle invigoration by reward anticipation is predicted by increased opportunity costs.
• This model makes predictions for a simplified neural implementation.

## 1 Introduction

The mammalian startle reflex is a protective postural change and eye blink response. It occurs in response to a suspected immediate blow to the head or upper body, as signalled by sudden noise, sharp movement, or touch (Yeomans et al., 2002). Similar protective reflexes are observed in non-mammalian species (Walters et al., 1981). Startle responses are extremely rapid – with a motor output delay of 5 ms (hindlimb in rats) to 10 ms (eye blink in humans) (Yeomans et al., 2002). Despite this, they can be modulated by the state of the environment. For example, after presentation of a stimulus previously conditioned to predict an aversive event (a conditioned stimulus, CS+), the startle reflex to a subsequent startle probe is increased (Brown et al., 1951) compared to startle reflex after a CS-, a phenomenon termed fear-potentiated startle. This is found in various species including aplysia (Walters et al., 1981), mice (Falls et al., 1997), rats (Chi, 1965; Davis and Astrachan, 1978), rhesus monkeys (Antoniadis et al., 2007) and humans (Grillon et al., 1991; Grillon and Davis, 1997; Spence and Runquist, 1958). While the underlying neural pathway is largely known in mammals (Rosen and Davis, 1988; Walker and Davis, 1997a), formal approaches to explain this observation have drawn on descriptive models of underlying psychological states (Lang et al., 1990) or neurobiological processes (Ramirez-Moreno and Sejnowski, 2012). Yet, these models neither explain the adaptive value of this behaviour nor can they accommodate all experimental observations. In particular, their predictions are in contradistinction to the pattern of startle modulation by reward and instructed attention commonly observed in humans (Sabatinelli et al., 2001; Skolnick and Davidson, 2002; Lipp et al., 1997, 1998). Here, we fill this lacuna with a formal account of the startle response in the framework of Bayesian decision theory (Körding, 2007). This provides a formal model of the startle response and its well established context sensitivity. Because the model is based upon Bayesian optimality principles it is normative. Furthermore, biologically plausible implementations suggest themselves by appeal to theories like predictive coding (Rao and Ballard, 1999).

The underlying idea of this model is that any organism must maximise its fitness. The objective of behaviour in a given situation is therefore to minimise any cost that reduce fitness. We make the simple assumption that the startle response per se is adaptive by protecting the organism from physical impact of a blow, and that startle response is more effective in doing so when it is more vigorous (Yeomans et al., 2002). Secondly and crucially, we assume that the organism assigns a common cause to physical manifestations of danger. That is to say, the organism infers from the likely presence of aversive events such as electric shocks used in experimental paradigms to the likely presence of other threats such as a blow to the head, because both are assumed to be manifestations of the same cause. This non-specificity is normative in environments in which manifestations of physical danger are correlated. To simplify presentation in the Results/Discussion, a blow is assumed to be either present or absent. This well reflects most experimental manipulations discussed in the paper. To account for more realistic biological scenarios, the appendix contains a generalisation for a continuous (scalar) blow magnitude which replicates all results from the discrete model.

## 2 Model

### 2.1 Model outline

When suspecting a blow, the objective of an adaptive organism is to minimise its impact on fitness, and we can quantify this impact using cost functions. We unpack total cost *C*_*TOT*_ into two types: costs of the blow *C*_*B*_, and costs of the startle response itself, *C*_*R*_. If startle response itself had no cost, the organism should always exhibit the largest possible startle magnitude, in order to minimise the cost of the blow; because this is not the case (Yeomans et al., 2002), there must be a cost to the response. Each of these two cost terms can be further unpacked into direct costs *C*_*d*_ and costs due to foregone opportunities *C*_*f*_. $C_{B,d}(r)$ quantifies the direct cost of a blow (e.g. tissue damage) which depends on the startle response magnitude *r* because startle is assumed to be more effective when it is more vigorous. $C_{R,d}(r)$ is the direct (metabolic) cost of the startle response which also depends on its magnitude as it consumes more energy when the response is more vigorous. Opportunity costs, on the other hand, are the potential benefits foregone due to interruption of ongoing behaviour, either through the startle response itself, $C_{R,f}(r)$, or because of the physical impact of the blow, $C_{B,f}(r)$. We assume all costs combine additively (assumption 1), and treat the blow *B* from the perspective of the agent as Bernoulli-distributed random variable – i.e. it will either occur or not. Success probability of this random variable is P(B|X), i.e. the probability of a blow *B*, given the current sensory input *X* which includes the startling stimulus *S*. According to decision theory, the organism needs to minimise total cost, which is the sum of startle response cost, and expected cost of the blow to the organism. Here ${\langle \cdot \rangle }_{P}$ denotes the expectation under P(B|X)

$$C_{\mathrm{TOT}}(r)=C_{R}(r)+{\langle C_{B}(r)\rangle }_{P}=C_{R}(r)+P(B|X)C_{B}(r)=[C_{R,d}(r)+C_{R,f}(r)]+P(B|X)[C_{B,d}(r)+C_{B,f}(r)].$$

### 2.2 Assumptions

#### 2.2.1 Assumption 1

*All costs combine additively and are fully known to the agent*.

This is the only assumption that bears on the structure of the model; the following assumptions constrain the behaviour of the model but not its structure.

#### 2.2.2 Assumption 2

*Increasing startle response reduces direct cost of a blow but not the probability of a blow*:

$$\frac{d}{\mathrm{dr}}C_{B}(r)<0,$$

P(B|r,X)=P(B|X).

#### 2.2.3 Assumption 3

*Changing the utility of foregone opportunities linearly scales the opportunity cost functions by a factor η*

$$C_{\mathrm{TOT}}^{⁎}(r)=C_{R,d}(r)+{\langle C_{B,d}(r)\rangle }_{P}+\eta C_{R,f}(r)+\eta {\langle C_{B,f}(r)\rangle }_{P}.$$

The change in opportunity cost thus only depends on the changing utility of the current action that the startle response, or the blow, would interrupt. This is based on the biological assumption that changing the utility of this action does not change the probability of performing it successfully.

#### 2.2.4 Assumption 4

*The relative increase in opportunity cost of the startle response is smaller than the relative decrease in opportunity cost of the blow when startle response is increased*:

$$|\frac{d}{\mathrm{dr}}C_{R,f}(r)|<|\frac{d}{\mathrm{dr}}C_{B,f}(r)|.$$

$$\frac{d}{\mathrm{dr}}C_{R,f}(r)>0.$$

$$\frac{d}{\mathrm{dr}}C_{B,f}(r)<0.$$

This assumption is required to explain the impact of increasing opportunity cost on startle response (Section 2.4). Globally this is biologically reasonable: a maximum magnitude startle response interrupts ongoing behaviour for a shorter time than a blow in the absence a startle response. Hence, the maximum opportunity cost of the startle is smaller than the maximum opportunity cost of the blow. Here, we need to stipulate that this relation is given locally over the entire range of startle responses and expected blow magnitudes to which the model is applied.

From this assumption, it follows that for all $r<r_{0}$:

$$C_{R,f}(r)+{\langle C_{B,f}(r)\rangle }_{P}>C_{R,f}(r_{0})+{\langle C_{B,f}(r_{0})\rangle }_{P}.$$

### 2.3 Impact of increasing blow probability

When we increase the blow probability P(B|X) to $P^{⁎}(B|X)$, the global minimiser for $C_{\mathrm{TOT}}^{⁎}$, $r_{0}^{⁎}$, must be larger than, or equal to, the current minimiser for *C*_*TOT*_, *r*_0_: $r_{0}^{⁎}\geq r_{0}$.

Proof Assume some $r<r_{0}$. We show that this cannot be a minimiser for $C_{\mathrm{TOT}}^{⁎}$. □

First, we expand the total cost for the new blow probability (star notation indicates the situation with increased blow probability)

$$C_{\mathrm{TOT}}^{⁎}(r)=C_{R}(r)+{\langle C_{B}(r)\rangle }_{P^{⁎}}=C_{R}(r)+P^{⁎}(B|X)/P(B|X){\langle C_{B}(r)\rangle }_{P}=C_{\mathrm{TOT}}(r)+(P^{⁎}(B|X)/P(B|X)-1){\langle C_{B}(r)\rangle }_{P}=C_{\mathrm{TOT}}(r)+{\mathrm{zC}}_{B}(r),$$

where $z=P^{⁎}(B|X)-P(B|X)$. Because *r*_0_ is a global minimiser, $C_{\mathrm{TOT}}(r)>C_{\mathrm{TOT}}(r_{0})$ for all $r\neq r_{0}$. Hence

$$C_{\mathrm{TOT}}(r)+{\mathrm{zC}}_{B}(r)>C_{\mathrm{TOT}}(r_{0})+{\mathrm{zC}}_{B}(r).$$

Now $P^{⁎}(B|X)>P(B|X)$ and therefore, z>0. Also, by Assumption 2, ${\mathrm{dC}}_{B}(r)/\mathrm{dr}<0$ and therefore $C_{B}(r)>C_{B}(r_{0})$ for $r<r_{0}$. Hence

$$C_{\mathrm{TOT}}(r_{0})+{\mathrm{zC}}_{B}(r)>C_{\mathrm{TOT}}(r_{0})+{\mathrm{zC}}_{B}(r_{0}).$$

Undoing the substitution, we obtain

$$C_{\mathrm{TOT}}(r_{0})+{\mathrm{zC}}_{B}(r_{0})=C_{\mathrm{TOT}}^{⁎}(r_{0}).$$

To summarise

$$r<r_{0}\Rightarrow C_{\mathrm{TOT}}^{⁎}(r)>C_{\mathrm{TOT}}^{⁎}(r_{0}).$$

This means that no $r<r_{0}$ can be a global miminiser for $C_{\mathrm{TOT}}^{⁎}$. Therefore the new global minimiser must be some $r_{0}^{⁎}\geq r_{0}$.

### 2.4 Impact of increasing cost of foregone opportunities

When we increase opportunity cost, the new global minimiser for $C_{\mathrm{TOT}}^{⁎}$, $r_{0}^{⁎}$, must be larger than, or equal to, the current minimiser for *C*_*TOT*_, *r*_0_: $r_{0}^{⁎}\geq r_{0}$.

Proof Assume $r<r_{0}$. We will show that this cannot be a minimiser for $C_{\mathrm{TOT}}^{⁎}$. □

First, we can expand the total cost for the situation with higher opportunity cost (indicated by star notation), using Assumption 3 with η>1:

$$C_{\mathrm{TOT}}^{⁎}(r)=C_{R,d}(r)+{\langle C_{B,d}(r)\rangle }_{P}+C_{R,f}^{⁎}(r)+{\langle C_{B,f}^{⁎}(r)\rangle }_{P}=C_{R,d}(r)+{\langle C_{B,d}(r)\rangle }_{P}+\eta (C_{R,f}(r)+{\langle C_{B,f}(r)\rangle }_{P})=C_{\mathrm{TOT}}(r)+(\eta -1)(C_{R,f}(r)+{\langle C_{B,f}(r)\rangle }_{P})>C_{\mathrm{TOT}}(r_{0})+(\eta -1)(C_{R,f}(r)+{\langle C_{B,f}(r)\rangle }_{P})>C_{\mathrm{TOT}}(r_{0})+(\eta -1)(C_{R,f}(r_{0})+{\langle C_{B,f}(r_{0})\rangle }_{P})=C_{\mathrm{TOT}}^{⁎}(r_{0}),$$

from Assumption 4, and because η>1. To summarise

$$r<r_{0}\Rightarrow C_{\mathrm{TOT}}^{⁎}(r)>C_{\mathrm{TOT}}^{⁎}(r_{0})$$

This means that no $r<r_{0}$ can be a global miminiser for *C*_*TOT*_. Therefore the new global minimiser must be some $r_{0}^{⁎}\geq r_{0}$.

### 2.5 Bayesian analysis of the fear-potentiated startle paradigm

If a sensory input *X* is comprised by a CS+ and a startle stimulus S, we have, by Bayes׳ theorem

$$P(B|X=\{S,\mathrm{CS}+\})=\frac{P(X=\{S,\mathrm{CS}+\}|B)P(B)}{P(X=\{S,\mathrm{CS}+\})}.$$

Under the simplifying assumption that the agent believes CS+ and S are independent: p(X={S,CS+})=p(S)p(CS+), and therefore

$$P(B|X=\{S,\mathrm{CS}+\})=\frac{P(S|B)P(\mathrm{CS}+|B)P(B)}{P(S)P(\mathrm{CS}+)}=\frac{P(S|B)P(B|\mathrm{CS}+)}{P(S)}.$$

In other words, the probability of a blow under the current sensory input depends on the probability of a blow given the CS+. This is the quantity that changes by fear conditioning.

Also

$$P(B|X=\{\neg S,\mathrm{CS}+\})=\frac{P(\neg S|B)P(B|\mathrm{CS}+)}{P(\neg S)}\approx 0,$$

because

P(¬S|B)≈0.

This means that if the CS+ is presented without the startle stimulus *S*, then the probability of a blow is close to zero, because the probability of no startle stimulus when there is an immediate blow is almost zero. The last equation reflects the biological observation that animals show no startle responses in the absence of startle stimuli, and from this it follows animals should not startle when the CS is presented on its own.

## 3 Results

We first consider the startle response on its own. According to decision theory, the organism needs to minimise the cost of the startle response, plus the expected cost of a blow: $C_{\mathrm{TOT}}=C_{R}+P(B|X)C_{B}$. Expected cost of a blow is the second term in the sum – the product of blow probability and blow cost. Crucially, if the blow probability p(B|X) becomes higher, the cost-minimising startle magnitude increases (Section 2.3). Hence, if a stimulus *X*_2_ is more likely to signify a blow than another stimulus *X*_1_, then $P(B|X_{2})>P(B|X_{1})$ - and therefore, the cost-minimising startle response is more vigorous for *X*_2_ than for *X*_1_. In biological terms, this predicts for example that a louder noise which is more likely to signify a blow also elicits a stronger startle response, because this is cost-minimising under the model, in line with experimental observations (Dawson et al., 2008).

How does the organism estimate p(B|X), the probability of a blow, given its sensory inputs? Is it fixed, learned by experience, or inferred from other available variables? This question can be addressed by analysing the fear-potentiated startle paradigm (Brown et al., 1951). Here, an animal is trained to associate a sensory stimulus (conditioned stimulus, CS+) with an aversive outcome (unconditioned stimulus, US). After this association is established, a startling sensory stimulus *S* is presented at some time during the CS+. As a rule, startle magnitude is higher in this situation than in the presence of a CS−which is predictive of US omission. In our model, this change in startle magnitude can only be explained if P(B|X={S,CS+})>P(B|X={S,CS−}). But this inequality could not arise if p(B|X) was fixed: the assignment of CS+ and CS− is entirely arbitrary. Hence, we can exclude this first possibility for establishing P(B|X). Secondly, if P(B|X) was learned by experience, the animal would have to experience the startling stimulus in the presence of the CS+ and CS− (i.e. {S,CS+} and {S,CS−}) at least once before a difference between p(B|X={S,CS+}) and p(B|X={S,CS−}) could arise. But training the CS–US association changes startle magnitude on the first startle trial after learning, in the absence of any previous experience with the combination of startle stimulus and CS. Hence, P(B|X) cannot be learned by experience. As a third possibility, we can use Bayes׳ theorem and unpack this probability into P(B|X)=P(X|B)P(B)/P(X). Formally, P(B|X) is a backward model of possible scenarios, given sensory input, and P(X|B) is a forward model of which sensory input to expect, given a particular scenario. Unpacking the backward model in this way, and rearranging terms, P(B|X={S,CS+}) depends on P(B|CS+) (Section 2.5). Hence, if the organism has learned that CS+ predicts US and assigns this US to a process also causing a threat *B*, this will increase P(B|CS+) and thereby increase P(B|X). In this case, the expected cost of the blow will increase, and hence, the cost-minimising startle response. In other words, startle potentiation after a CS+ that predicts a physical impact (fear-potentiated startle) normatively arises from Bayesian decision theory. At the same time, one can show that startle magnitude will be near-zero (i.e. no startle response will be elicited) if the CS+ is presented without the startle-eliciting stimulus (Section 2.5).

This model also makes another prediction. If we write P(B|X)=P(X|B)P(B)/P(X), then the prior probability *P*(*B*) does not depend on current sensory input *X* and can be estimated from past experience. Hence, if *P*(*B*) is increased before the startle probe occurs, startle response can be increased in the absence of “fear-eliciting” sensory input at the moment of startle elicitation. Experiments that manipulate the prior probability *P*(*B*) have accumulated evidence in favour of this prediction. Startle potentiation is seen in trace fear conditioning (Burman and Gewirtz, 2004), contextual fear conditioning (Campeau et al., 1991; Grillon and Davis, 1997), after prior exposure to foot shocks (Hitchcock et al., 1989), and during instructed fear in humans (Grillon et al., 1993). All these manipulations increase *P*(*B*) before the startle probe, *X*, occurs. Further, our model explains a number of related phenomena which are often framed in psychological or ethological terms. For rats, a crepuscular (twilight-active) species, bright light is associated with danger – and also increases startle magnitude (Walker and Davis, 1997b), while for diurnal humans, darkness is associated with danger and increases startle magnitude (Grillon et al., 1997). In humans, imagination of negative events (Vrana and Lang, 1990; Vrana, 1995; Cook et al., 1991) and the anticipation of negative pictures (Sabatinelli et al., 2001) increase startle. Two seconds or longer after onset of negatively valenced pictures in humans, startle to visual and auditory probes is increased (Sabatinelli et al., 2001; Vrana et al., 1988; Bradley et al., 1990; Codispoti et al., 2001). In our model, it is not the psychological valence of these pictures that matters but their property of predicting physical danger, i.e. increasing *P*(*B*). In line with this, negative picture viewing only increases startle if pictures are rated high in subjective arousal (Cuthbert et al., 1996) or explicitly depict situations of physical threat (Bradley et al., 2001). On the other hand, a few seconds after onset of positively valenced images (Sabatinelli et al., 2001; Vrana et al., 1988; Bradley et al., 1990; Codispoti et al., 2001),startle magnitude is reduced – in our model this follows from a smaller prior probability of a blow.

Up to here, our model makes similar predictions to a psychological “motivational priming” model (Lang et al., 1990) according to which motivational states amplify compatible reflexes and reduce incompatible ones. In this model, startle reflex is seen to be compatible with negative but not positive motivational state; hence it is amplified by punishments but reduced by gains.

**Fig. 1:**
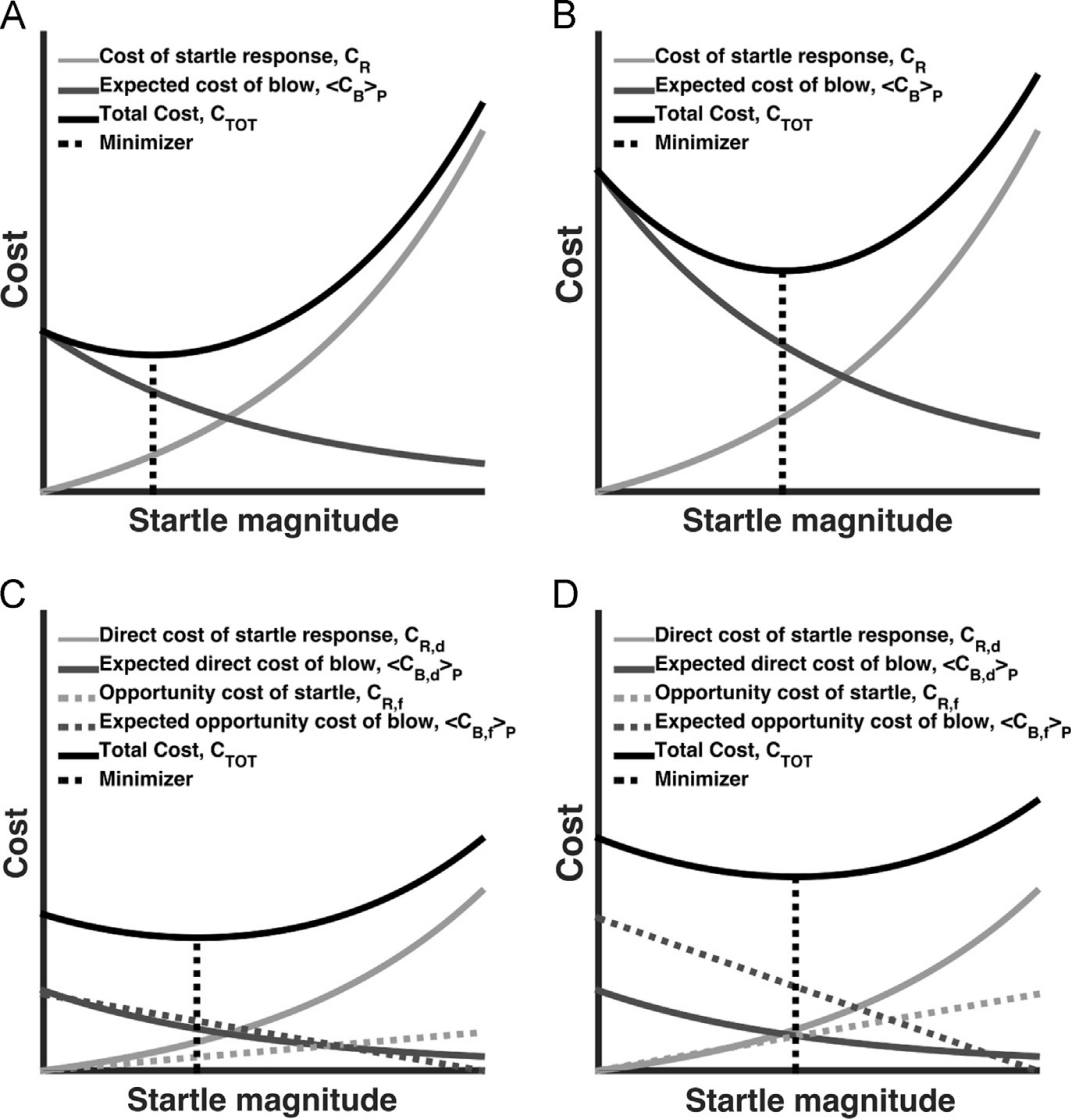
Examples for cost functions and change of parameters. (A) Startle cost (light grey) increases with increasing startle magnitude, while expected cost of a blow (dark grey) decreases with increasing startle magnitude. They are added into a total cost function (black), and this is minimized to determine optimal startle magnitude. (B) Increasing blow probability scales the expected cost of blow and increases the minimiser for the total cost function – hence, optimal startle magnitude is increased. Blow probability given sensory input can, by Bayes theorem, be increased via increased prior probability of a blow (see text). (C) Opportunity costs are potential benefits, foregone due to the startle response (dotted light grey) or due to the blow (dotted dark grey). They are combined with the direct costs to give a total cost function (black). (D) Increasing the potential benefits scales the opportunity cost function. This shifts the minimising startle magnitude towards higher values – provided that the opportunity costs of startle have shallower slope than the opportunity costs of the blow, a biological meaningful assumption (see text).

However, our predictions crucially diverge from this model when it comes to the impact of reward anticipation and top-down (instructed) attention. In a motivational priming model, both reward anticipation and collection of reward imply motivational states which are incompatible with startle reflex and should therefore reduce startle magnitude. In order to analyse these situations in our model, we need to consider opportunity costs. Clearly, a startle response interrupts the organism such it might forego a reward at the same time – this imposes an opportunity cost. However, the impact of physical danger might interrupt the organism for much longer such that benefit of foregone opportunities will typically be higher. Crucially, by Assumption 4, the relation between startle magnitude and startle opportunity cost, $C_{R,f}$, is less steep than the relation between startle magnitude and opportunity cost due to the blow, $C_{B,f}$. This is illustrated in Fig. 1 and implies that increasing opportunity cost increases the optimal startle magnitude if probability of a blow is constant (Section 2.4). Anticipation of reward in humans increases opportunity cost – because the rewarding event might be missed – and hence can increase cost-minimising startle magnitude. In line with this prediction, anticipating positive pictures or financial gains in humans increases startle magnitude (Skolnick and Davidson, 2002; Sabatinelli et al., 2001). Here, predictions from our model are entirely different from previous models which do not take into account opportunity cost. Equally, there is an opportunity cost to missing a stimulus that one is instructed to attend. There is experimental evidence that in this situation, startle magnitude is increased over and above modality-specific effects of attentional gain (Lipp et al., 1998, 1997). Note that these predictions depend on a constant probability of a blow. However we have argued that positively valenced stimuli decrease the prior probability of a blow, *p*(*B*). If they also increase opportunity cost, optimal startle magnitude increases only if the increased opportunity cost outweighs the impact of reduced danger, and this is difficult to establish in the aforementioned studies. Much clearer evidence for our model comes from an experiment on food stimuli. For satiated subjects, food stimuli decrease the prior probability of danger and thus decrease optimal startle magnitude. For food-deprived subjects, however, these visual signals predict reward (the possibility of eating food) and increase opportunity cost. This may increase optimal startle response. Indeed, this dissociation has experimentally been observed (Drobes et al., 2001).

While the startle potentiation circuit is well-characterised (Walker and Davis, 1997a), there is not much research that could help elucidate the computations embedded in this modulatory circuit. However, one study suggests that it is not simply *P*(*B*) or P(B|CS+) which is being signalled. Davis and Astrachan found that conditioning rats with a footshock as US increases startle magnitude after the CS+ across the board, in keeping with previous evidence and with our model. However, training with a very high-magnitude footshock US increased startle less than conditioning with medium-magnitude footshock US (Davis and Astrachan, 1978). This can be framed in an extended model (see Appendix) which takes into account the scalar blow magnitude *b*. After a CS+ predicting a very high magnitude footshock, the organism would, in this model, expect a stronger blow than after a CS+ signalling a medium-blow footshock, both with the same probability. The observed pattern of startle potentiation can arise if Assumption 2 from the appendix is violated, i.e. if there is a range of very high *b* over which startle is less effective at reducing cost than at lower values of *b*. Biologically this could mean that a startle response is a less efficient response for very strong than for medium blow magnitudes.

Crucially, any modulatory pathway should in principle encode the full probability distribution over blow magnitudes, p(B=b), to be incorporated with the bivariate cost function $C_{B}(s,b)$. Yet, this does not seem to be the case. In the aforementioned study, during extinction in the medium-footshock group, startle magnitude consistently decreased, as would be predicted in our model by a progressive decrease in *P*(*B*) under extinction. However, in the high-footshock group, startle magnitude first increased during extinction and then decreased again. This is not optimal behaviour: if startle response is inefficient to protect from a strong blow that is very likely, it does not become more efficient for the same strong blow when it is less likely. This finding can only be understood if an expectation of blow magnitude, rather than its probability distribution, is combined with the cost function. This could occur in the modulatory pathway encoding *p*(*B*) but also in the startle-eliciting node encoding p(B|X). Encoding an expectation rather than a probability distribution is sparse. Such simplified computations have also been observed in motor decisions (Fleming et al., 2013). This algorithm will produce close approximations to optimal startle magnitude over regions where Assumption 2 from the appendix is fulfilled. Otherwise, this simplified architecture comes at the expense of performance in this circuit. Surprisingly little is known about startle modification under reward-induced states in animals, such that no predictions for neural implementation of opportunity cost minimisation is currently possible.

## 4 Discussion

In this paper, we presented a normative approach to startle modulation, by formalising the consequences of a startle response as costs, and analysing the cost-minimising behaviour. Under the assumption that startle is adaptive by reducing the impact of a potential blow, we find that the general structure of the model is able to capture startle modulation by different startle probes. Crucially, using a Bayesian approach to unpack the probability of a blow, this model can account for the well-established phenomenon of fear-potentiated startle (Brown et al., 1951) in which a startle probe is combined with a CS+ that predicts an aversive outcome. The model generalises this situation to other situations in which the prior probability of a blow is increased from the perspective of an organism, but in which no “fear-eliciting” stimulus is presented at the time of the startle probe. Importantly, by considering opportunity costs, the model makes the prediction that anticipation of rewards, and instructed attention, can increase startle reflex. This is in keeping with experimental observations and in contradistinction to previous models which could not account for this phenomenon (Lang et al., 1990). To summarise, almost all behavioural observations on startle modification follow normatively from the presented model. Because the model formalises selective pressures in terms of costs, it also explains why this behaviour evolved.

Mathematical models in neuroscience can be broadly classified according to Marr׳s three levels of analysis (Marr and Poggio, 1976): computational – the problem to be solved, algorithmic – by which algorithm the problem is solved, implementation – how this is biophysically encoded in neural circuits. Our model is on a computational level: we show which behaviour solves the problem of cost-minimisation under some constraints but not by which algorithm or in which neural circuits the organism solves this problem on-line. However, one can make some predictions about neural implementation. An important conceptual difference between our model and previous psychological models (Lang et al., 1990) is that in our model, startle modification does not require a central motivational state – the required computations can be implemented locally (LeDoux, 2014). More specifically, startle reflex is instantiated in a minimal brain stem circuit, and modulatory influences impact on this circuit. These modulatory influences are well-characterised for potential punishments. Input from the basolateral nucleus of the amygdala (BLA) appears to be crucial for modulation in danger states (Walker and Davis, 1997a). This information is relayed to the BLA from central nucleus of the amygdala in conditioned fear (Walker and Davis, 1997a), and from bed nucleus of the stria terminalis in bright light exposure of rats, and anticipatory anxiety (Walker et al., 2003). Intracerebroventricular infusion of the stress hormone corticotropin-releasing factor, CRF, increases startle magnitude in rats (Swerdlow et al., 1986; Liang et al., 1992). Physiological CRF release is seen under acute threat, so this hormone might signal an increased prior probability of a blow as well, although it is not yet known whether this impact is also mediated by the BLA. To summarise, these results strongly suggest that the BLA is crucial in signalling modulatory influences to the brain stem startle circuit. The observation that decreasing the probability of very high-magnitude footshock in rats can increase, rather than decrease, startle magnitude (Davis and Astrachan, 1978) appears to follow from a simplified neural architecture in which a modulatory pathway signals blow expectation rather than a full probability distribution of blows. This circuit produces near-optimal results under the condition that higher startle magnitude is more efficient at reducing blow cost, but becomes clearly suboptimal if that condition is not fulfilled.

Besides explaining existing observations, the model also makes additional testable predictions. Specifically, startle magnitude is assumed to be monotonically related to the probability of a blow, which is easy to test experimentally by varying the probability of danger - for example by probabilistic punishment schedules in a fear conditioning task. Also, startle magnitude is assumed to monotonically relate to opportunity costs.

Our model makes few assumptions. One assumption that bears on the structure of the model is that costs combine additively. The remaining assumptions impact the behaviour of the model and can be analysed within the given model structure. We have already highlighted one example in which Assumption 2 appears to be violated under extreme circumstances, and it should be straightforward to test the range of startle and blow magnitudes under which the assumptions are fulfilled.

A crucial biological assumption we make in the case of fear-potentiated startle is that the organism assigns aversive events such as an electric shock to a common cause also predicting a threatening blow. This assumption in itself is normative in environments in which different manifestations of physical threat are correlated. It remains to be shown whether this generalisation also occurs for danger manifestations that are typically not correlated in biological environments, for example threat of predation and threat of conspecific attack. This would educe whether startle potentiation is an evolved mechanism adaptive in highly complex environments, or whether its unspecificity is due to constraints on the complexity of the neural system. Such constraints are apparent in simple species, e.g. aplysia, in which fear-potentiated startle can be found.

Finally, the model focuses on determining the optimal startle magnitude independent of other actions. It is possible that startle response is selected from a larger action repertoire, and in this case there might be interactions between the cost functions related to startle, and to other actions. Selection between, for example, startling and freezing may explain the aforementioned observation that during fear conditioning with very high electric shocks, startle potentiation is smaller than with medium shocks.

In the case of fear-potentiated startle, our model bases optimal startle magnitude on Bayesian inference. This fits into a class of theories about the “Bayesian Brain” postulating that the brain in general uses probabilistic inference and stores forward models and prior probabilities to compute optimal behaviour using Bayes׳ theorem (Pouget et al., 2013). In terms of neurobiological implementation, this form of the model lends itself to implementation through predictive coding (Rao and Ballard, 1999; Dayan and Hinton, 1996). This is important because there is a growing literature on such implementation schemes, one of them being active inference. In this scheme, the cost functions above are casted as prior beliefs, and then the Bayes optimal expression of a startle response can be expressed as a pure inference problem of the sort solved by Bayesian filtering. The functional anatomy reviewed above may then be understandable in terms of Bayesian belief updating of the sort associated with hierarchical message passing in the brain (Bastos et al., 2012).

To summarise, the presented model furnishes a novel perspective upon the context sensitivity of startle reflex and arranges empirical findings in a computational framework. By doing so, it furnishes an exemplary approach to bridging an empirical and theoretical gap between human emotion psychology, and animal neuroscience. Thus, it may pave the way towards a cross-species and computational perspective upon emotion neuroscience.

## Figures and Tables

**Table A1:** Variables and notation in the model for scalar blow magnitude.

Variable	Explanation
*r*	Scalar magnitude of startle response
B=b∈B	Continuous random variable, denoting scalar magnitude of blow to the organism
*X*	Sensory information at the time of the startle response
p(b|X)	Probability density function over blow magnitudes, given sensory information
$p^{\left(1\;..\;n\right)}(B=b|X)$	Set of p.d.f.s describing all possible scenarios of conditional blow expectations
$p^{⁎}(B=b|X)$	P.d.f for which ${\langle B\rangle }_{p^{⁎}}>{\langle B\rangle }_{p}$
${\langle \cdot \rangle }_{p}$	Expectation under p(B|X): $\int_{b\in B}\mathrm{db}(\cdot )p(B|X)$
${\langle \cdot \rangle }_{p^{⁎}}$	Expectation under $p^{⁎}(B|X)$: $\int_{b\in B}\mathrm{db}(\cdot )p^{⁎}(B|X)$
$C_{R,d}(r)$	Direct metabolic cost of startle response
$C_{R,f}(r)$	Cost of opportunities foregone due to the startle response
$C_{B,d}(r,b)$	Direct physical cost of the blow
$C_{B,f}(r,b)$	Cost of opportunities foregone due to the blow
$C_{R}(r)=C_{R,d}(r)+C_{R,f}(r)$	Overall startle response cost
$C_{B}(s,b)=C_{B,d}(r,b)+C_{B,f}(r,b)$	Overall blow cost
$C_{\mathrm{TOT}}(s)=C_{R}(r)+{\langle C_{B}(r,b)\rangle }_{p}$	Total cost

## Appendix A Model for scalar blow magnitude

### A.1 Assumptions

#### A.1.1 Assumption 1

*All costs combine additively and are fully known to the agent* (Table A1).

#### A.1.2 Assumption 2

*Startle is more effective in reducing expected cost at high expected blow magnitude than at lower expected blow magnitude*:

$${\langle B\rangle }_{p^{⁎}}>{\langle B\rangle }_{p}\Rightarrow \frac{d}{\mathrm{dr}}{\langle C_{B}(r)\rangle }_{p^{⁎}}<\frac{d}{\mathrm{dr}}{\langle C_{B}(r)\rangle }_{p},$$

$$\frac{d}{\mathrm{dr}}{\langle C_{B}(r)\rangle }_{p}<0,$$

where the star indicates the situation with higher expected blow magnitude. This generalises Assumption 2 in the main text. It constrains the combinations of cost functions and probability distributions that are allowed in the model. Consider the more general relation

$$\frac{\partial }{\partial r}C_{B,d}(r,b)<0,$$

$$\frac{\partial }{\partial b}\frac{\partial }{\partial r}C_{B,d}(r,b)<0,$$

which states that startle is more effective at higher startle magnitudes, and is more or equally effective in reducing cost at higher blow magnitudes. Assumption 2 follows from these relations for example in the following two special cases:

• a cost function derivative $\left(\partial /\partial r)C_{B}(r,b\right)$ concave and increasing in *b*, combined with any set of probability distributions for *B*
• a set of p.d.f.s $p^{\left(1\;..\;n\right)}(B|X)$ with the same shape and variance but different mean (e.g. Gaussian p.d.f.s)

From Assumption 2, it follows for all $r<r_{0}$:

$${\langle C_{B}(r)\rangle }_{p^{⁎}}-{\langle C_{B}(r_{0})\rangle }_{p^{⁎}}>{\langle C_{B}(r)\rangle }_{p}-{\langle C_{B}(r_{0})\rangle }_{p}.$$

Rearranging this inequality, we obtain

$${\langle C_{B}(r)\rangle }_{p^{⁎}}-{\langle C_{B}(r)\rangle }_{p}>{\langle C_{B}(r_{0})\rangle }_{p^{⁎}}-{\langle C_{B}(r_{0})\rangle }_{p}.$$

In other words, the effect of increasing expected blow magnitude is larger at low than at high startle magnitudes.

#### A.1.3 Assumption 3

*Changing the utility of foregone opportunities linearly scales the opportunity cost functions by a factor η*

$$C_{\mathrm{TOT}}^{⁎}(r)=C_{R,d}(r)+{\langle C_{B,d}(r)\rangle }_{p}+\eta C_{R,f}(r)+\eta {\langle C_{B,f}(r)\rangle }_{p}.$$

This generalises Assumption 3 from the main text.

#### A.1.4 Assumption 4

*The relative increase in opportunity startle cost is smaller than the relative decrease in expected opportunity cost of the blow when startle magnitude is increased by a certain amount*

$$|\frac{d}{\mathrm{dr}}C_{R,f}(r)|<|\frac{d}{\mathrm{dr}}{\langle C_{B,f}(r)\rangle }_{p}|.$$

$$\frac{d}{\mathrm{dr}}C_{R,f}(r)>0.$$

$$\frac{d}{\mathrm{dr}}\langle C_{B,f}{(r)\rangle }_{p}<0.$$

Again, this generalises Assumption 4 from the main text. From this assumption, it follows that for all $r<r_{0}$:

$$C_{R,f}(r)+{\langle C_{B,f}(r)\rangle }_{p}>C_{R,f}(r_{0})+{\langle C_{B,f}(r_{0})\rangle }_{p}.$$

### A.2 Impact of increasing expected blow magnitude

When we increase the expected blow magnitude, the global minimiser for $C_{\mathrm{TOT}}^{⁎}$, $r_{0}^{⁎}$, must be larger than the current minimiser for *C*_*TOT*_, *r*_0_. Proof Assume $r<r_{0}$. We can expand the total cost for the new expected blow magnitude (star notation indicates the situation with increased expected blow magnitude):

$$C_{\mathrm{TOT}}^{⁎}(r)=C_{R}(r)+{\langle C_{B}(r)\rangle }_{p^{⁎}}=C_{R}(r)+{\langle C_{B}(r)\rangle }_{p}+{\langle C_{B}(r)\rangle }_{p^{⁎}}-{\langle C_{B}(r)\rangle }_{p}=C_{\mathrm{TOT}}(r)+{\langle C_{B}(r)\rangle }_{p^{⁎}}-{\langle C_{B}(r)\rangle }_{p}.\;□$$

*r*_0_ is a global minimiser, so $C_{\mathrm{TOT}}(r)>C_{\mathrm{TOT}}(r_{0})$ for all $r\neq r_{0}$. Hence

$$C_{\mathrm{TOT}}(r)+{\langle C_{B}(r)\rangle }_{p^{⁎}}-{\langle C_{B}(r)\rangle }_{p}>C_{\mathrm{TOT}}(r_{0})+{\langle C_{B}(r)\rangle }_{p^{⁎}}-{\langle C_{B}(r)\rangle }_{p}.$$

Because of Assumption 2:

$$C_{\mathrm{TOT}}(r_{0})+{\langle C_{B}(r)\rangle }_{p^{⁎}}-{\langle C_{B}(r)\rangle }_{p}>C_{\mathrm{TOT}}(r_{0})+{\langle C_{B}(r_{0})\rangle }_{p^{⁎}}-{\langle C_{B}(r_{0})\rangle }_{p}.$$

To summarise

$$r<r_{0}\Rightarrow C_{\mathrm{TOT}}^{⁎}(r)>C_{\mathrm{TOT}}^{⁎}(r_{0}).$$

This means that no $r<r_{0}$ can be a global miminiser for *C*_*TOT*_. Therefore the new global minimiser must be some $r_{0}^{⁎}\geq r_{0}$.

### A.3 Impact of increased opportunity cost

When we increase opportunity cost, the new global minimiser for $C_{\mathrm{TOT}}^{⁎}$, $r_{0}^{⁎}$, must be larger than the current minimiser for *C*_*TOT*_, *r*_0_. Proof Assume $r<r_{0}$. We can expand the total cost for the new opportunity cost (star notation indicates the situation with increased opportunity cost), using Assumption 3 with η>1

$$C_{\mathrm{TOT}}^{⁎}(r)=C_{R,d}(r)+{\langle C_{B,d}(r)\rangle }_{p}+C_{R,f}^{⁎}(r)+{\langle C_{B,f}^{⁎}(r)\rangle }_{p}=C_{\mathrm{TOT}}(r)+(\eta -1)C_{R,f}(r)+(\eta -1){\langle C_{B,f}(r)\rangle }_{p}>C_{\mathrm{TOT}}(r_{0})+(\eta -1)(C_{R,f}(r)+{\langle C_{B,f}(r)\rangle }_{p}).>C_{\mathrm{TOT}}(r_{0})+(\eta -1)(C_{R,f}(r_{0})+{\langle C_{B,f}(r_{0})\rangle }_{p})=C_{\mathrm{TOT}}^{⁎}(r_{0}),\;□$$

From Assumption 4, and because η>1. To summarise

$$r<r_{0}\Rightarrow C_{\mathrm{TOT}}^{⁎}(r)>C_{\mathrm{TOT}}^{⁎}(r_{0}).$$

This means that no $r<r_{0}$ can be a global miminiser for *C*_*TOT*_. Therefore the new global minimiser must be some $r_{0}^{⁎}\geq r_{0}$.
